# Comparative assessment of ensemble and machine learning models for susceptibility mapping of 2024 Wushi earthquake-triggered landslides

**DOI:** 10.1371/journal.pone.0362380

**Published:** 2026-11-10

**Authors:** Yutong Wu, Junpeng Huang, Wenqian Li, Xiangfeng Jia, Yaning Li, Zhiyi Wang

**Affiliations:** 1 School of Geology and Mining Engineering, Xinjiang University, Urumqi, China; 2 Key Laboratory of Green and Efficient Mining and Ecological Restoration in High-Altitude Arid Regions of Xinjiang, Urumqi, China; China Construction Fourth Engineering Division Corp. Ltd, CHINA

## Abstract

Coseismic landslide susceptibility mapping plays a critical role in disaster risk mitigation, infrastructure protection, and territorial spatial planning, particularly in tectonically active mountain belts such as the Tianshan Mountains. Conventional machine learning models often suffer from overfitting and inadequate generalization capability due to their homogeneous structures and inflexible modeling strategies. In this study, targeting the coseismic landslides triggered by the 2024 Ms 7.1 Wushi earthquake in the Biedieli River basin, a landslide inventory comprising 904 events was compiled and randomly partitioned into training (70%) and validation (30%) datasets. Nine conditioning factors pertaining to topography, geology, environment, and seismicity were selected, including elevation, slope angle, slope aspect, curvature, lithology, distance to faults, distance to rivers, distance to roads, and peak ground acceleration (PGA). The predictive performance of an ensemble learning model, LightGBM, was compared against three benchmark machine learning models, i.e., artificial neural network (ANN), decision tree (DT), and naive Bayes (NB). Multicollinearity diagnosis and factor importance ranking were conducted using variance inflation factor (VIF) and correlation attribute evaluation (CAE), respectively. The model performance was validated and compared via the area under the receiver operating characteristic curve (AUC) and several statistical metrics including accuracy and F_1_-score. The results indicate that elevation (AM = 0.6296), PGA (AM = 0.5810), and distance to roads (AM = 0.4934) are the primary determinants of landslide occurrence. The LightGBM model achieved the best overall predictive performance on the validation dataset, with an AUC of 0.961 and the highest accuracy (0.890) and F_1_-score (0.895), followed by ANN (AUC = 0.957, accuracy = 0.876, F_1_-score = 0.876), DT (AUC = 0.947, accuracy = 0.856, F_1_-score = 0.860), and NB (AUC = 0.919, accuracy = 0.862, F_1_-score = 0.869). From the training dataset to the validation dataset, the AUC decay of LightGBM was merely 2.6%, indicating good generalization capability. The high and very high susceptibility zones account for 10.51% to 19.43% of the study area across the four models, predominantly concentrated in the mid-elevation valley zones (2036.5–2959 m), steep slopes exceeding 40°, areas with PGA greater than 0.48 g, and the vicinity of the Maidan fault zone. The susceptibility maps produced in this study can provide a scientific basis for post-earthquake emergency response, hazard risk assessment, and disaster mitigation in the Tianshan seismic belt.

## 1 Introduction

Landslides are among the most common geological hazards worldwide, and in tectonically active mountainous regions, earthquake-induced coseismic landslides often result in severe casualties and economic losses [1,2]. Coseismic landslides not only directly destroy infrastructure such as roads and villages but may also block rivers, form dammed lakes, and trigger cascading secondary disasters [3]. The Tianshan orogenic belt, the largest intracontinental rejuvenated orogen in Asia, has been shaped by the ongoing collision between the Indian and Eurasian plates since the Cenozoic, combined with intense endogenic and exogenic geological processes coupled with river incision and glacial erosion, which together produce steep topography and rapidly evolving geomorphic features in the Tianshan region [4]. Moreover, this area is characterized by strong tectonic activity and frequent earthquakes [5], making it an ideal region for studying coseismic landslides. On January 23, 2024, the Wushi Ms 7.1 earthquake struck the southern Tianshan, with a focal depth of 22 km and an epicenter located on a steep bank along the middle reaches of the Biedieli River, resulting in 3 fatalities and 5 injuries [6]. A large number of coseismic landslides triggered by this earthquake are densely distributed along both sides of the Biedieli River valley, posing serious threats to road traffic, residential safety, and the operation of infrastructure such as the Biedieli Port Highway, thus urgently requiring a regional coseismic landslide susceptibility assessment.

Currently, quantitative methods for coseismic landslide susceptibility assessment mainly include deterministic approaches based on physical-mechanical models and statistical methods based on data-driven techniques [7,8]. The deterministic approach, represented by the Newmark cumulative displacement method, is grounded in rigid-block sliding theory and evaluates failure probability by calculating permanent displacement of slopes under seismic loading, thus possessing clear physical significance [9]. However, this method requires high-quality geotechnical mechanical parameters (e.g., cohesion, internal friction angle, density) and detailed ground-motion inputs, and its simplified assumptions make it difficult to capture complex nonlinear relationships, limiting its applicability in vast mountainous areas with scarce basic data [10]. In recent years, machine learning models have been widely adopted for coseismic landslide susceptibility assessment due to their powerful nonlinear fitting capabilities, such as backpropagation neural network [11], Random Forest [12], support vector machine [13], and decision tree [14]. However, most existing studies focus on a limited number of mainstream algorithms. Distinct discrepancies exist in the principles and architectures of different models, whose predictive performance is strongly controlled by regional geo‑environmental conditions. Few investigations have addressed model adaptability for the alpine‑cold mountainous environment of the Tianshan Mountains. The Tianshan region differs from well-studied coseismic landslide areas such as the eastern margin of the Tibetan Plateau, the Himalayas, and the Apennines in several fundamental aspects. Climatically, the Tianshan region is characterized by an extremely arid and cold continental climate, with annual precipitation of approximately 180 mm and strong freeze-thaw cycling, in contrast to the monsoon-dominated and precipitation-rich Tibetan Plateau and Himalayan regions [15]. Tectonically, the Tianshan is an intracontinental rejuvenated orogen rather than a plate-boundary orogen, resulting in distinct patterns of fault activity and seismic energy release. Geomorphologically, the combination of river incision and glacial erosion produces steep, deeply dissected topography with limited vegetation cover and sparse field data, whereas the eastern Tibetan Plateau and the Apennines benefit from denser monitoring networks and more extensive field records [16]. These differences imply that machine learning models trained in well-studied regions may not be directly transferable to the Tianshan, and that region-specific model evaluation is necessary. Furthermore, machine learning models commonly suffer from overfitting and insufficient generalization ability [7].

Ensemble learning methods, such as Bagging and Boosting, which construct and combine predictions from multiple base learners, have been proven to be effective strategies for significantly improving model generalization and enhancing robustness [17]. Nevertheless, their performance depends on the selection of base learners, tuning of ensemble parameters, and data characteristics of the study area [18]. LightGBM, a Boosting-based ensemble model, iteratively builds decision trees through a gradient boosting framework, with each new tree focusing on correcting the residuals of the previous one; it employs leaf-wise growth and gradient-based one-side sampling (GOSS) techniques, enabling efficient processing of high-dimensional features and effective control of overfitting [19]. Nevertheless, the application of this model in coseismic landslide susceptibility assessment remains relatively limited, and its predictive performance in the high-altitude cold mountainous Tianshan region has yet to be tested. To justify model selection beyond exhaustive enumeration, four models were chosen to represent distinct algorithm paradigms: LightGBM (Boosting ensemble), ANN (artificial neural network, neural network), DT (decision tree, single-tree baseline), and NB (Naive Bayes, probabilistic generative classifier). Mainstream alternatives such as random forest (RF), XGBoost, gradient boosting decision tree (GBDT), and support vector machine (SVM) were not separately included because they either belong to the same broad paradigm as the selected models or have been widely examined in previous coseismic landslide studies [20]. The necessity of LightGBM lies in its leaf-wise growth, GOSS sampling, and exclusive feature bundling (EFB) technique, which are expected to balance computational efficiency and overfitting control for the moderately sized, highly clustered landslide dataset in the Biedieli River basin. Compared with RF, LightGBM is expected to achieve higher accuracy by sequentially correcting residuals; compared with XGBoost and GBDT, it is expected to be more efficient and less prone to overfitting under limited samples; compared with SVM, it avoids kernel-parameter tuning and scales better to high-dimensional nonlinear features. The expected advantages and limitations of the four models in this region are as follows: LightGBM should perform best in accuracy and generalization but is less interpretable; ANN can capture nonlinearity but may be constrained by the limited sample size and simple architecture [21]; DT provides a transparent baseline but may over-partition susceptible zones and is noise-sensitive; NB is efficient for small samples but its conditional-independence assumption may be violated by correlated conditioning factors [22]. By comparing these four fundamentally different models using the same dataset and a unified evaluation framework, we aim to identify the optimal model for the study area and reveal the applicability differences among various algorithms in the high-altitude cold mountainous environment of the Tianshan.

Therefore, this study takes the coseismic landslides in the Biedieli River basin triggered by the 2024 Wushi Ms 7.1 earthquake as the research object. A detailed coseismic landslide inventory was compiled, and nine conditioning factors were selected, including elevation, slope angle, slope aspect, curvature, lithology, distance to faults, distance to rivers, distance to roads, and peak ground acceleration (PGA). Then, the contribution of each factor using frequency ratio (FR), correlation attribute evaluation (CAE), and variance inflation factor (VIF) were analyzed. Furthermore, we compared the predictive performance for coseismic landslide susceptibility between the ensemble learning model (LightGBM) and three single-machine-learning models (ANN, DT, and NB). This study is expected to provide a scientific basis for coseismic landslide hazard prevention and mitigation in the Tianshan seismic zone and in regions with analogous geological and environmental settings.

## 2 Study area

The Biedieli River Basin, located in the western segment of the South Tianshan Mountains, is a major tributary of the Tuoshigan River (Fig 1). Its geographical coordinates range from 78°15′ to 78°40′ E and 41°10′ to 41°35′ N. The basin exhibits a narrow, NW-SE elongated shape, covering an area of approximately 412 km^2^. The terrain is characterized by high mountains in the northwest and low elevations in the southeast, with altitudes ranging from 1,729 m to 4,804 m above sea level. The study area features severe surface dissection, steep mountainous slopes, and significant topographic relief, representing a landscape of mid-high mountains and river valleys. The study area experiences a mid-temperate, plateau, and arid climate, characterized by cold temperatures, insufficient thermal energy, large diurnal temperature variations, uneven precipitation, and unstable snow cover. The mean annual precipitation is approximately 180 mm, with intense rainfall events mainly occurring in summer (June to August). Occasionally, short-duration rainstorms trigger flash floods.

**Fig 1:**
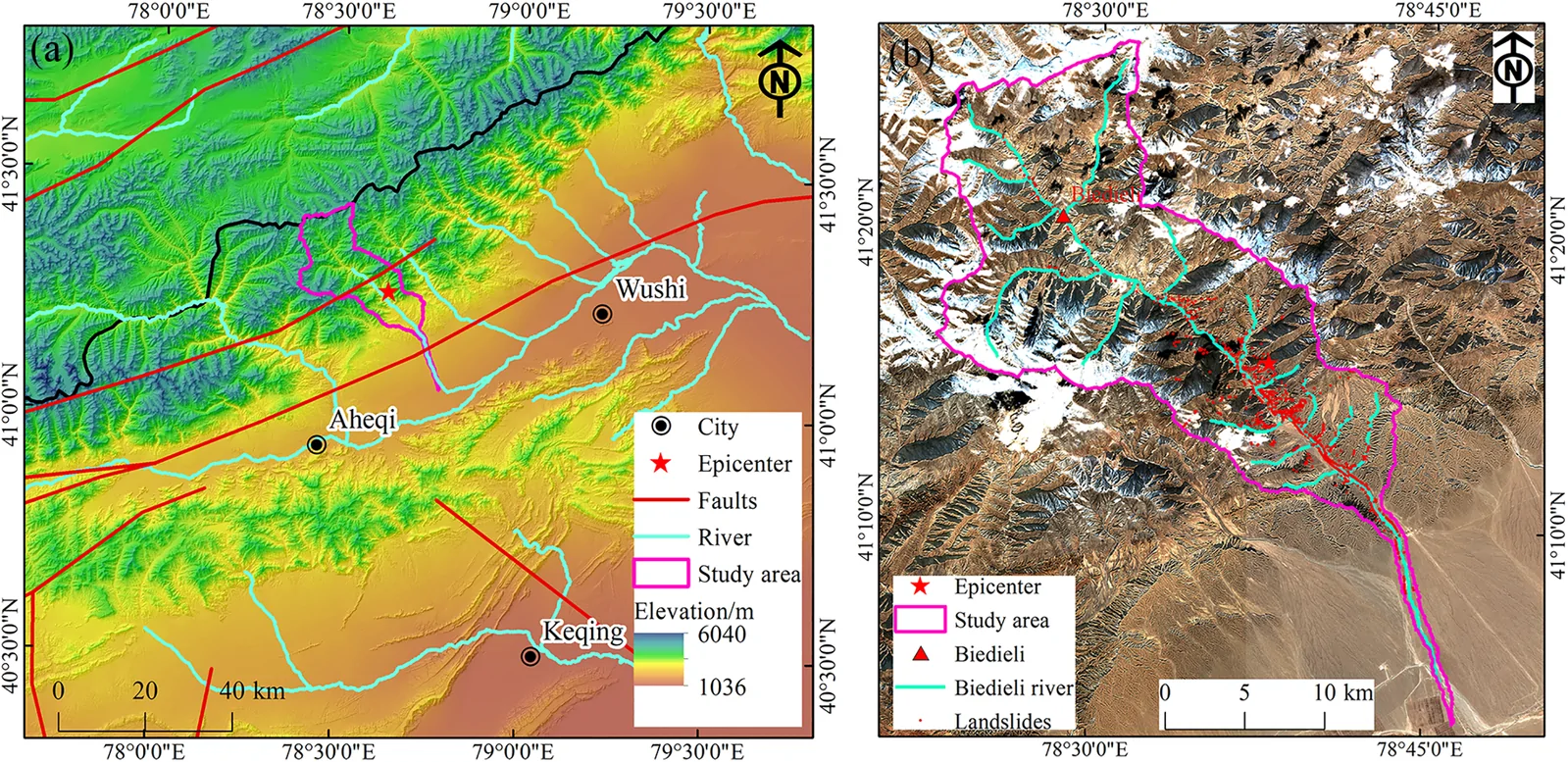
The geographical location of Biedieli River Basin. (a) Topographic map derived from the SRTM DEM (30 m resolution, https://earthexplorer.usgs.gov/), (b) Wushi earthquake-triggered landslide inventory overlaid on Sentinel-2 imagery (10 m resolution, https://dataspace.copernicus.eu/).

Since the Cenozoic, the continuous collision between the Indian and Eurasian plates has led to the reactivation and uplift of the South Tianshan, accompanied by its northward thrusting toward the Tarim Basin [23]. Multiple parallel NEE-SWW-trending thrust faults have developed in the piedmont zone of the South Tianshan. These imbricate thrusts propagate from north to south, causing the Tianshan block to subduct southward along a deep detachment fault onto the Precambrian crystalline basement of the Tarim Basin. The study area is located in the Wushi Depression, a transitional zone between the Kalpin and Kuqa thrust belts in the southwestern Tianshan. This region is characterized by extremely active neotectonics and seismicity. The middle and lower reaches of the Biedieli River Basin are composed of Paleogene, Neogene, and Quaternary strata, while the upper reaches consist of Devonian and Carboniferous strata.

On January 23, 2024, at 02:09 Beijing time, the Wushi Ms 7.1 earthquake struck Wushi County, Xinjiang, with a focal depth of 22 km. The epicenter (41.26° N, 78.63° E) is located on a steep slope along the middle reaches of the Biedieli River. The earthquake occurred near the Maidan Fault, a NEE-trending fault extending approximately 400 km, which serves as the boundary fault between the South Tianshan and the Tarim Basin. The Maidan Fault dips to the NE at moderate to high angles (30°–80°) and is characterized by thrusting with a strike-slip component [24]. The Wushi earthquake is the first Ms 7 + event in the Tianshan seismic belt since the 1992 Susamyr Ms 7.3 earthquake. The epicentral intensity reached IX, and the earthquake caused three fatalities and five injuries. This combination of steep topography, active tectonics, and strong seismicity creates a highly favorable environment for landslide initiation. Together, these characteristics make the Biedieli River Basin a representative case for coseismic landslide susceptibility research in the Tianshan seismic belt. First, the basin lies directly within the epicentral area of the 2024 Wushi Ms 7.1 earthquake, with the epicenter located on the middle-reach bank of the river, providing a rare opportunity to examine coseismic landsliding under near-field strong ground motion. Second, the basin integrates the key controlling conditions typical of the Tianshan region: steep topography shaped by river incision and glacial erosion, active thrust faulting associated with the Maidan Fault, widely developed river terraces composed of loose Quaternary deposits, and sparse vegetation under an arid and cold climate. Third, the basin hosts the Biedieli Port Highway, so that the landslide susceptibility results have direct implications for infrastructure safety and post-earthquake emergency response.

## 3 Materials and methods

The framework for coseismic landslide susceptibility mapping using machine learning and ensemble learning models in this study is illustrated in Fig 2. This framework consists of three main steps. Step 1: Construction of the coseismic landslide database, which includes establishing a landslide inventory based on field surveys and remote sensing interpretation, selecting optimal conditioning factors through multicollinearity analysis and correlation attribute evaluation, and partitioning the dataset into training and validation subsets. Step 2: Four models (ANN, DT, LightGBM, and NB) are employed to generate landslide susceptibility maps. Step 3: The performance of each model is evaluated and compared using objective quantitative metrics.

**Fig 2:**
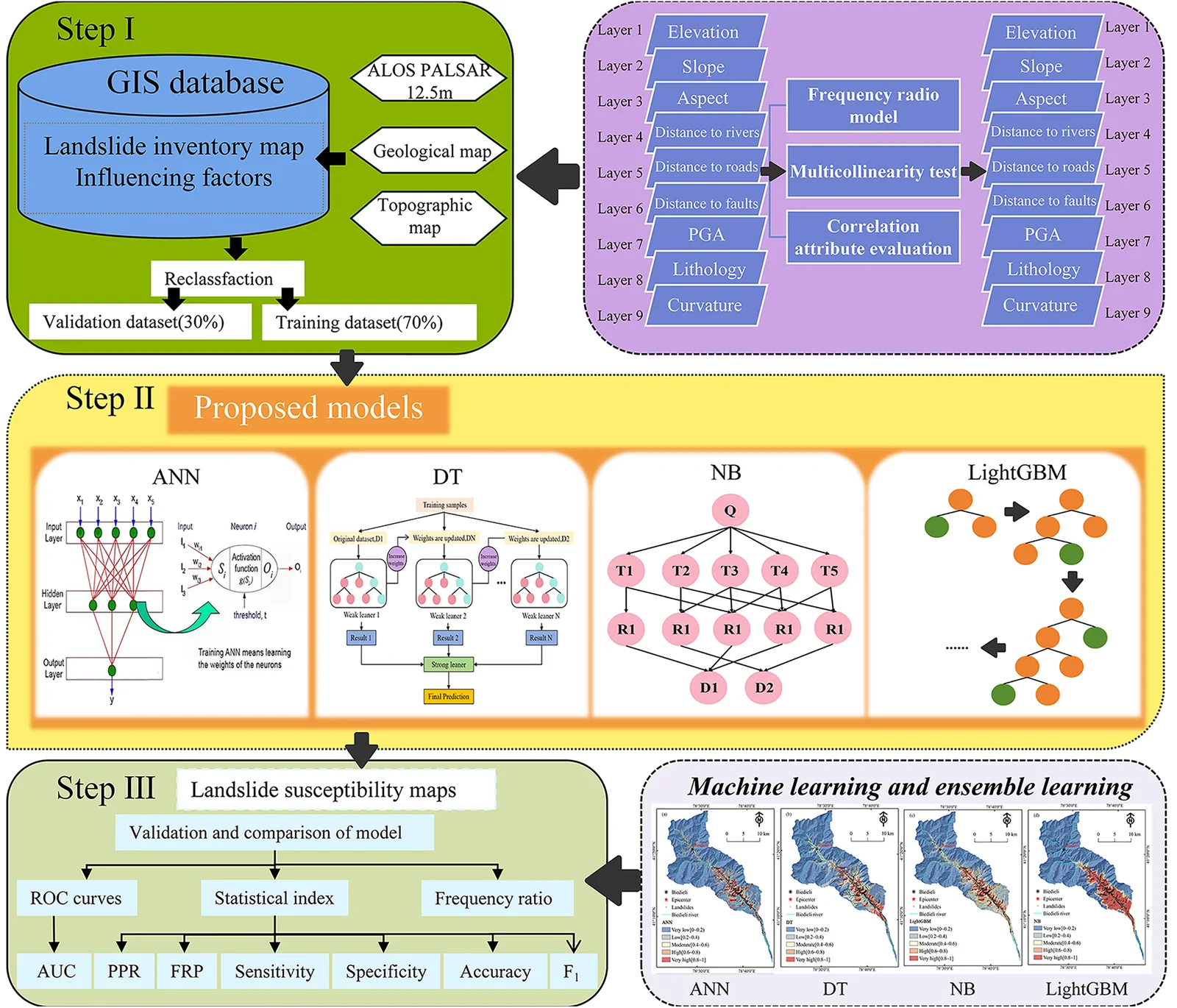
Flowchart of machine learning and ensemble learning models for coseismic landslide susceptibility assessment.

### 3.1 Coseismic landslide inventory

Following the 2024 Wushi Ms 7.1 earthquake, a coseismic landslide inventory for the Biedieli River Basin was established through a combination of high-resolution remote sensing image interpretation and field surveys. The remote sensing imagery used includes post-earthquake Jilin-1 (0.5 m resolution), Gaofen-7 (0.7 m resolution), and Gaofen-2 (0.8 m resolution) images, as well as pre-earthquake high-resolution Google Earth and Sentinel-2 images. Landslides were identified based on the following criteria: (1) presence of clear landslide boundaries (e.g., head scarps, flanks, or depositional lobes); (2) direct temporal association with the earthquake, excluding landslides triggered by rainfall or human activities; and (3) a minimum area of 50 m^2^. Field surveys were conducted from April 22–24, 2024, including a detailed field investigation and unmanned aerial vehicle (UAV) surveys along the Biedieli River, to verify the mapped landslide inventory. Field site access was approved by the Natural Resources Bureau of Wushi County, and all fieldwork was conducted in compliance with the relevant regulations.

A total of 904 coseismic landslides were identified in the study area. The total landslide area is approximately 2.65 km^2^, with individual landslide areas ranging from 54.32 m^2^ to 74,360.25 m^2^ and a mean area of 2,928.84 m^2^. Statistical analysis of landslide areas shows that 75% of the landslides are smaller than 2,500 m^2^ and 50% are smaller than 1,000 m^2^, indicating that small landslides dominate the inventory. The predominant landslide types include shallow debris slides, rock slides, and rockfalls (Fig 3). Spatially, the landslides are mainly distributed along both sides of the Biedieli River valley, concentrated near the Maidan fault zone, with the epicentral area located within this dense distribution zone.

**Fig 3:**
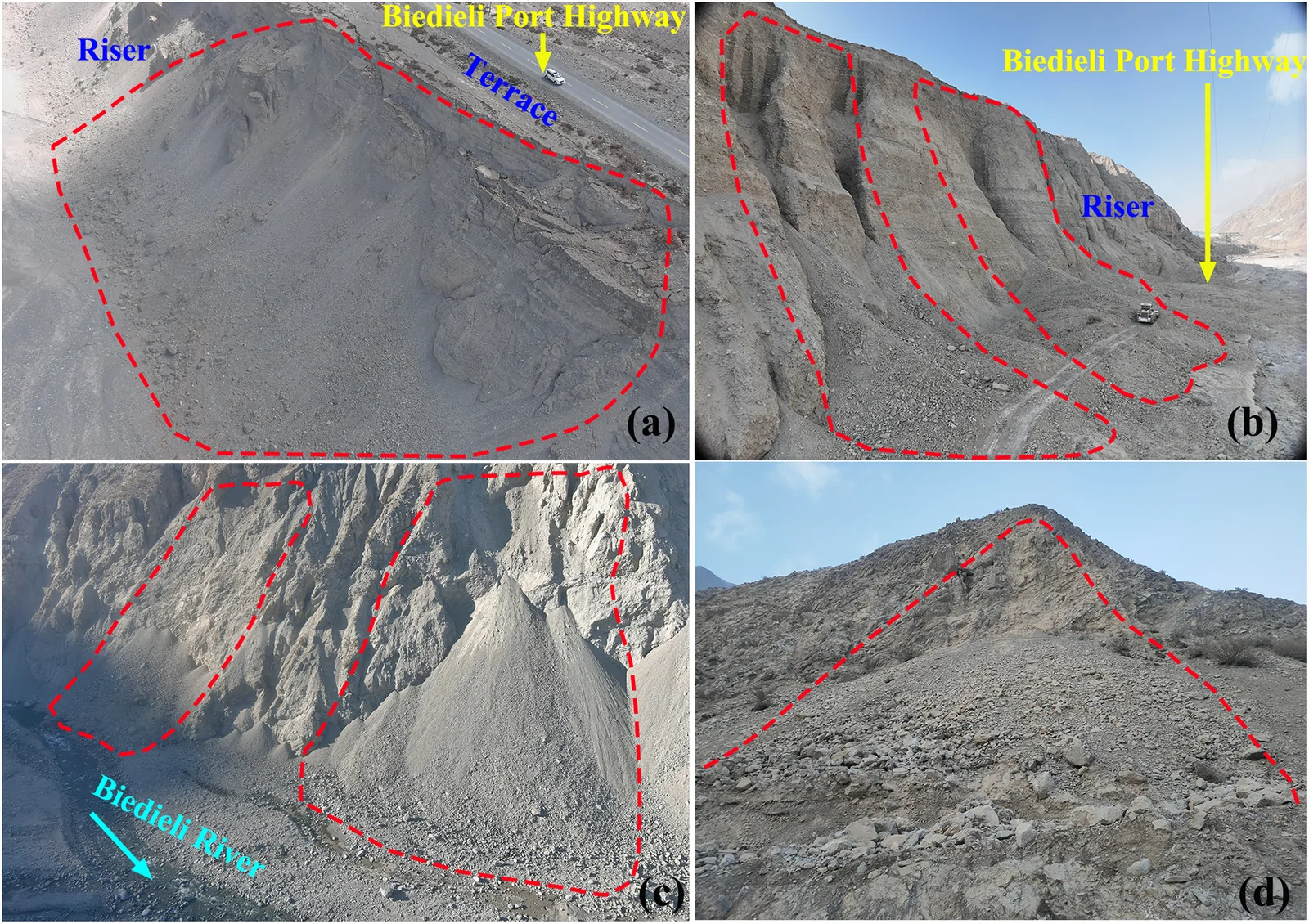
Field photographs of typical coseismic landslides. (a) and (b) shallow debris slides on terraces, (c) rock slides, and (d) rockfalls.

In addition, for model training and validation, a point-based sampling strategy was adopted. Each mapped landslide polygon was represented by its centroid as a landslide point, and non-landslide points were randomly sampled from areas outside the landslide polygons, with a number equal to that of the landslide points, to ensure a balanced dataset for model training. The sampled non-landslide points were further visually checked against high-resolution remote sensing imagery to exclude locations with ancient landslides, obvious human disturbance, or other potentially unstable features. The resulting dataset was then randomly divided into a training dataset (70%) and a validation dataset (30%). The 70/30 ratio is widely adopted in landslide susceptibility studies, as it balances adequate model training with reliable evaluation on an independent validation subset [7].

### 3.2 Coseismic landslide conditioning factors

Coseismic landslide occurrence is controlled by a complex combination of topographic, geological, environmental, and seismic factors. Based on previous studies of coseismic landslides (e.g., [6]) and the specific characteristics of the Biedieli River Basin, nine conditioning factors were selected for landslide susceptibility mapping: elevation, slope angle, aspect, curvature, lithology, distance to faults, distance to rivers, distance to roads, and peak ground acceleration (PGA). These factors have been widely employed and proven effective in previous coseismic landslide susceptibility studies [11,25]. These factors can be categorized into continuous factors (e.g., elevation, slope angle, aspect, curvature, distance to faults, distance to rivers, distance to roads, PGA) and categorical factors (lithology). The data sources for all factors are summarized in Table 1. All continuous factors were resampled to a uniform spatial resolution of 12.5 m × 12.5 m and transformed to the same coordinate system using ArcGIS 9.2 software. The categorical factor (lithology) was encoded as numerical values for model input.

**Table 1:** Coseismic landslide conditioning factors and data sources used in this study.

Factors	Type	Sources
Elevation/m	Continuous	ALOS PALSAR DEM 12.5m, https://vertex.daac.asf.alaska.edu/
Slope angle/°	Continuous	Derived from ALOS PALSAR DEM
Aspect	Continuous	Derived from ALOS PALSAR DEM
Curvature	Continuous	Derived from ALOS PALSAR DEM
Lithology	Categorical	1:200000 geological map of the China Geological Survey, https://www.cgs.gov.cn/
Distance to faults/m	Continuous	
Distance to rivers/m	Continuous	Derived from ALOS PALSAR DEM
Distance to roads/m	Continuous	National Platform for Common Geospatial Information Services of China, https://www.tianditu.gov.cn/
PGA/g	Continuous	ShakeMap published by the USGS (2024), https://earthquake.usgs.gov

#### 3.2.1 Topographic factors.

Elevation influences landslide susceptibility by affecting slope stability, vegetation cover, and human activities [11]. The elevation map was derived from the ALOS PALSAR DEM (12.5 m resolution), ranging from 1,729 m to 4,804 m (Fig 4a). Slope angle is one of the most critical factors controlling landslide initiation. Steeper slopes experience higher shear stress, making them more prone to failure [26]. The slope map (Fig 4b) was calculated from the DEM. In the study area, slope angles range from 0° to 77.87°. Aspect influences landslide occurrence by modulating solar radiation, precipitation patterns, and wind exposure [27]. The aspect map (Fig 4c) was derived from the DEM and categorized into eight classes: north (N), northeast (NE), east (E), southeast (SE), south (S), southwest (SW), west (W), and northwest (NW). Curvature reflects the convergence or divergence of surface water flow, affecting pore water pressure and slope stability. Curvature values (Fig 4d) were calculated from the DEM, with values of <−0.05 representing concave slopes, > 0.05 values representing convex slopes, and −0.05 ~ 0.05 representing flat surfaces.

**Fig 4:**
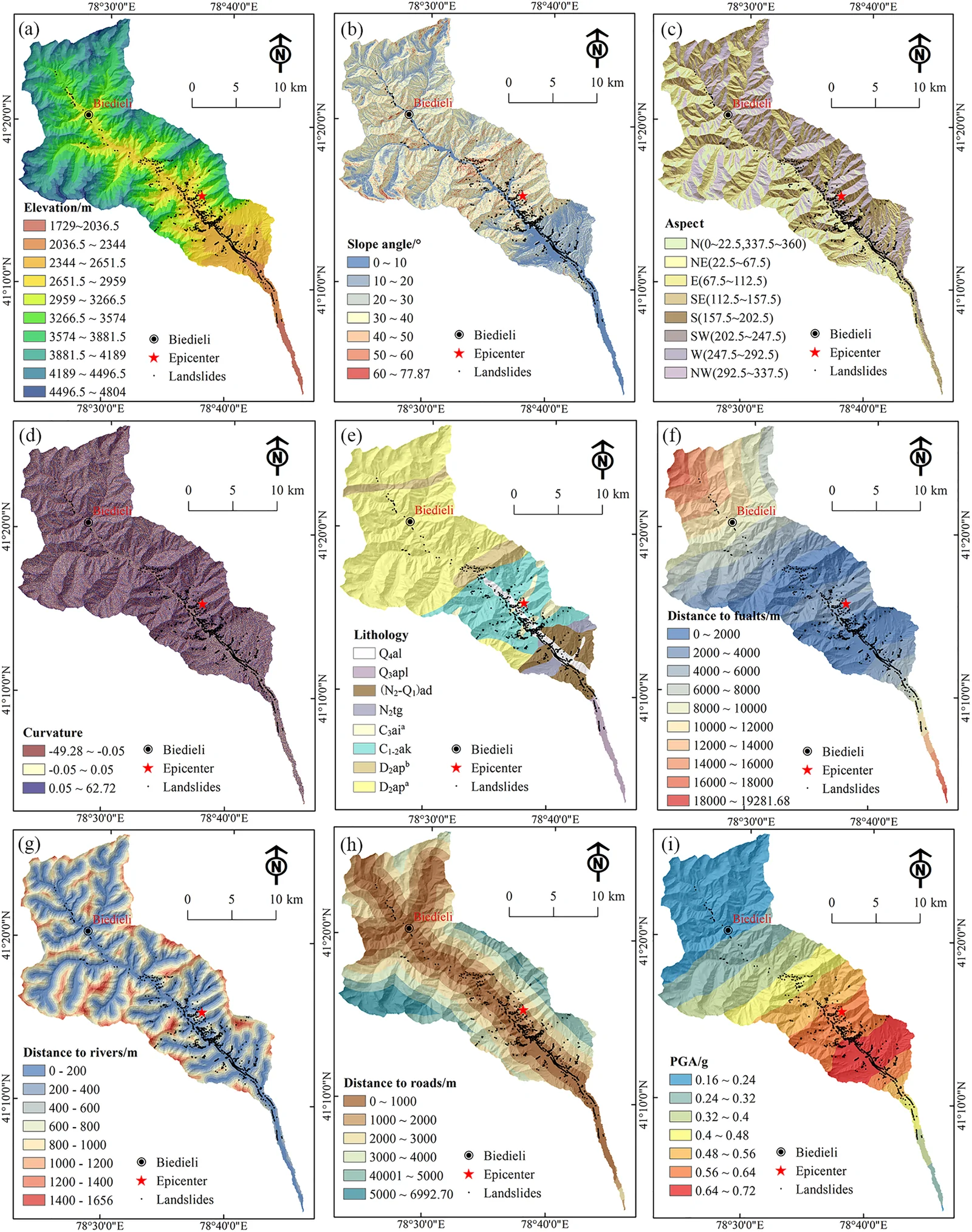
Coseismic landslide conditioning factors. (a) Elevation, (b) slope angle, (c) slope aspect, (d) curvature, (e) lithology, (f) distance to faults, (g) distance to rivers, (h) distance to roads, and (i) peak ground acceleration (PGA).

#### 3.2.2 Geological factors.

Lithology is a key geological factor that controls the mechanical properties and permeability of slope materials. The lithological map (Fig 4e) was derived from the 1:200,000 geological map of the China Geological Survey. According to the geological age and rock strength characteristics, the lithology of the study area was divided into 8 groups: Q_4_al, alluvial deposits; Q_3_apl, alluvial-diluvial deposits; (N_2_-Q_1_)ad, conglomerate with sandstone lens; N_2_tg, conglomerate, calcareous sandstone, quartz siltstone; C_3_ai^a^, limestone and sandstone; C_1-2_ak, sandstone, siltstone, limestone and gypsum; D_2_ap^b^, limestone and tuff conglomerate; D_2_ap^a^, phyllite, siltstone and limestone. Distance to faults is an important factor for coseismic landslide susceptibility, as faults control the spatial distribution of strong ground motion during earthquakes. The distance to faults (Fig 4f) was calculated using the Euclidean distance tool in ArcGIS 9.2 based on the fault map derived from the geological map.

#### 3.2.3 Environmental factors.

Distance to rivers influences slope stability through riverbank erosion and changes in pore water pressure. The river network was extracted from the ALOS PALSAR DEM, and the Euclidean distance was calculated (Fig 4g). Distance to roads reflects the impact of human activities, such as road construction and traffic vibrations, on slope stability. The road network data used in this study refer to the Biedeli Port Highway, derived from the National Platform for Common Geospatial Information Services of China. The Euclidean distance to this road was calculated using ArcGIS 9.2 (Fig 4h).

#### 3.2.4 Seismic factor.

PGA is the primary seismic factor controlling the initiation of coseismic landslides. PGA measures the intensity of ground shaking during an earthquake, with higher PGA values generally corresponding to a greater likelihood of landslide occurrence. The PGA map for the 2024 Wushi Ms 7.1 earthquake was obtained from the ShakeMap published by the United States Geological Survey (USGS, 2024) (Fig 4i). In the study area, PGA values range from 0.16 g to 0.72 g.

### 3.3 Multicollinearity analysis and factor selection

#### 3.3.1 Multicollinearity analysis.

Before inputting the conditioning factors into the machine learning models, a multicollinearity analysis was performed to detect strong correlations among the nine selected factors [28]. High collinearity between factors can lead to redundant information, model instability, and reduced interpretability of the model output. In this study, the variance inflation factor (VIF) was used to quantify the degree of multicollinearity. VIF measures how much the variance of a regression coefficient is inflated due to collinearity with other factors [29]. It is calculated as:


$$\begin{array}{c} VIF_{i}=1/\left(1-R_{i}^{\text{2}}\right) \end{array}$$ (1)

where *R*_*i*_^*2*^ is the coefficient of determination obtained by regressing the *i*-th factor against all other factors. A VIF value greater than 10 is generally considered indicative of problematic collinearity.

#### 3.3.2 Correlation attribute evaluation.

To evaluate the predictive power of each conditioning factor with respect to landslide occurrence, a correlation attribute evaluation (CAE) was conducted. The CAE method evaluates the importance of each factor by measuring its correlation with the landslide presence variable [30]. For each conditioning factor, an average merit (AM) value is calculated, which reflects the relative contribution of that factor to the prediction of landslide occurrence. A higher AM value indicates a stronger correlation between the factor and landslide occurrence, suggesting a greater predictive power.

### 3.4 Frequency ratio analysis

The frequency ratio (FR) is a bivariate statistical method used to quantitatively assess the spatial relationship between each conditioning factor and landslide occurrence. The FR is defined as the ratio of the percentage of landslides occurring in a given factor class to the percentage of area occupied by that class [31]:


$$\begin{array}{c} FR=\left(N_{ij}/N\right)/\left(A_{ij}/A\right) \end{array}$$ (2)

where *N*_*ij*_ is the number of landslide pixels in factor class *j* of factor *i*, *N* is the total number of landslide pixels in the study area, *A*_*ij*_ is the area of factor class *j* of factor *i*, and *A* is the total area of the study area.

An FR value greater than 1 indicates a positive correlation between the factor class and landslide occurrence, meaning that landslides are more likely to occur in that class. Conversely, an FR value less than 1 indicates a negative correlation, suggesting a lower likelihood of landslide occurrence.

In this study, the FR was calculated for each class of the nine conditioning factors using the landslide inventory and factor maps. For the continuous factors, appropriate classification intervals were determined before FR calculation. Specifically, with the exception of curvature, all continuous factors were classified using the equal interval method.

### 3.5 Machine learning models

#### 3.5.1 Artificial neural network (ANN).

ANN is a computational model inspired by the structure and function of biological neural networks. An ANN consists of an input layer, one or more hidden layers, and an output layer, each containing multiple neurons connected by weighted edges. The network learns the relationship between input features and output targets through a process of forward propagation and backpropagation [32]. In this study, the ANN model was implemented in Python 3.11 using the MLPClassifier in scikit-learn (Version 1.4.0), with the following optimized hyperparameters: a single hidden layer containing 64 neurons (hidden_layer_sizes = (64,)), an L2 regularization parameter alpha = 0.1, and an initial learning rate learning_rate_init = 0.01.

#### 3.5.2 Decision tree (DT).

DT is a non-parametric supervised learning method that predicts the value of a target variable by learning simple decision rules inferred from the data features [33]. It partitions the feature space into rectangular regions, each corresponding to a decision rule, and assigns a predicted value to each region. The DT model is easy to interpret and can handle both continuous and categorical data. In this study, the DT model was implemented using the DecisionTreeClassifier in scikit-learn, with the following optimized hyperparameters: Gini impurity as the splitting criterion (criterion = gini), a maximum tree depth of 8 (max_depth = 8), and a minimum of 20 samples per leaf (min_samples_leaf = 20).

#### 3.5.3 Light gradient boosting machine (LightGBM).

LightGBM is a gradient boosting ensemble method with decision trees (CART) as its base learner [34]. Unlike single classifiers, LightGBM sequentially trains multiple decision trees, where each new tree corrects the errors of the previous ones, and the final prediction is obtained by summing the weighted contributions of all trees [35]. LightGBM incorporates two key techniques: Gradient-based One-Side Sampling (GOSS) and Exclusive Feature Bundling (EFB). GOSS focuses on under-fitted samples, while EFB reduces feature dimensionality, both improving training efficiency. Unlike traditional boosting methods that grow trees level-wise, LightGBM adopts a leaf-wise growth strategy, leading to faster convergence and lower memory usage. In this study, LightGBM was implemented using the LGBMClassifier in the official LightGBM Python library (Version 4.3.0), with the default gradient boosting decision tree (GBDT) boosting type (boosting_type = ‘gbdt’) and binary log-loss (objective = ‘binary’). Hyperparameters were optimized via RandomizedSearchCV with five-fold cross-validation on the training dataset, over the following search space: n_estimators ∈ [20, 100], num_leaves ∈ [4, 15], max_depth ∈ [2, 4], learning_rate ∈ [0.01, 0.1], min_child_samples ∈ [40, 150], reg_alpha, reg_lambda ∈ [0.1, 10], subsample ∈ [0.6, 0.9], and colsample_bytree ∈ [0.5, 0.8]. The final optimized hyperparameters were: n_estimators = 100, learning_rate = 0.1, num_leaves = 4, max_depth = 4, min_child_samples = 80, subsample = 0.8, colsample_bytree = 0.8, and reg_alpha = reg_lambda = 0.1.

#### 3.5.4 Naive bayes (NB).

NB classifier is a probabilistic machine learning model based on Bayes’ theorem with the assumption of conditional independence among features [36]. Despite its simplicity, the NB model has been widely used in landslide susceptibility studies due to its computational efficiency and robustness to irrelevant features [37]. In this study, the NB model was implemented using the GaussianNB in scikit-learn, with the default hyperparameter var_smoothing = 1e-9 and no additional tuning.

### 3.6 Model validation and evaluation

To objectively compare and validate the goodness-of-fit and predictive power of the four models, a comprehensive evaluation was conducted using statistical indices derived from the confusion matrix and the receiver operating characteristic (ROC) curve. The statistical indices include the positive predictive rate (PPR, also known as precision), the false positive rate (FPR), the true positive rate (TPR, also known as sensitivity or recall), specificity, accuracy, and the F_1_-score. PPR represents the proportion of correctly predicted landslides among all samples predicted as landslides, reflecting the reliability of landslide prediction; FPR measures the proportion of non-landslide samples that are incorrectly predicted as landslides; TPR (sensitivity) reflects the ability to correctly identify landslides; specificity reflects the ability to correctly identify non-landslides; accuracy measures the overall proportion of correct predictions; and the F_1_-score is the harmonic mean of PPR and TPR, providing a balanced measure when precision and recall are both considered. Higher values of accuracy, PPR, TPR, specificity, and F_1_-score indicate better model performance [38].

In addition, the ROC curve was plotted to evaluate the overall discriminative ability of each model. In the ROC curve, the x-axis represents the FPR, and the y-axis represents the TPR. The area under the ROC curve (AUC) was then calculated to quantify the overall performance, with AUC values closer to 1 indicating better discriminative ability [26]. The relevant formulas are as follows [39]:


$$\begin{array}{c} PPR=Precision=\frac{TP}{TP+FP} \end{array}$$ (3)

$$\begin{array}{c} FPR=\frac{FP}{FP+TN} \end{array}$$ (4)

$$\begin{array}{c} Sensitivity=TPR=\frac{TP}{TP+FN} \end{array}$$ (5)

$$\begin{array}{c} Specificity=\frac{TN}{FP+TN} \end{array}$$ (6)

$$\begin{array}{c} Accuracy=\frac{TP+TN}{TP+FP+TN+FN} \end{array}$$ (7)

$$\begin{array}{c} F_{\text{1}}=\frac{2*Sensitivity*\;PPR}{Sensitivity+PPR} \end{array}$$ (8)

$$\begin{array}{c} AUC=\int_{\text{0}}^{\text{1}}TPR\left(FPR\right)\text{d}FPR \end{array}$$ (9)

where TP (true positives) and TN (true negatives) are the numbers of correctly classified landslide and non-landslide instances, respectively; FP (false positives) and FN (false negatives) are the numbers of misclassified landslide and non-landslide instances, respectively.

## 4 Results

### 4.1 Factors analysis

#### 4.1.1 Frequency ratio analysis.

Regarding elevation, the FR results show that the classes of 2,036.5–2,344 m (FR = 8.91), 2,344–2,651.5 m (FR = 4.52), and 2,651.5–2,959 m (FR = 2.27) all have FR values greater than 1, indicating that these are high-susceptibility zones for landsliding (Fig 5a). Among them, the class of 2,344–2,651.5 m contains 41.4% of all landslides.

**Fig 5:**
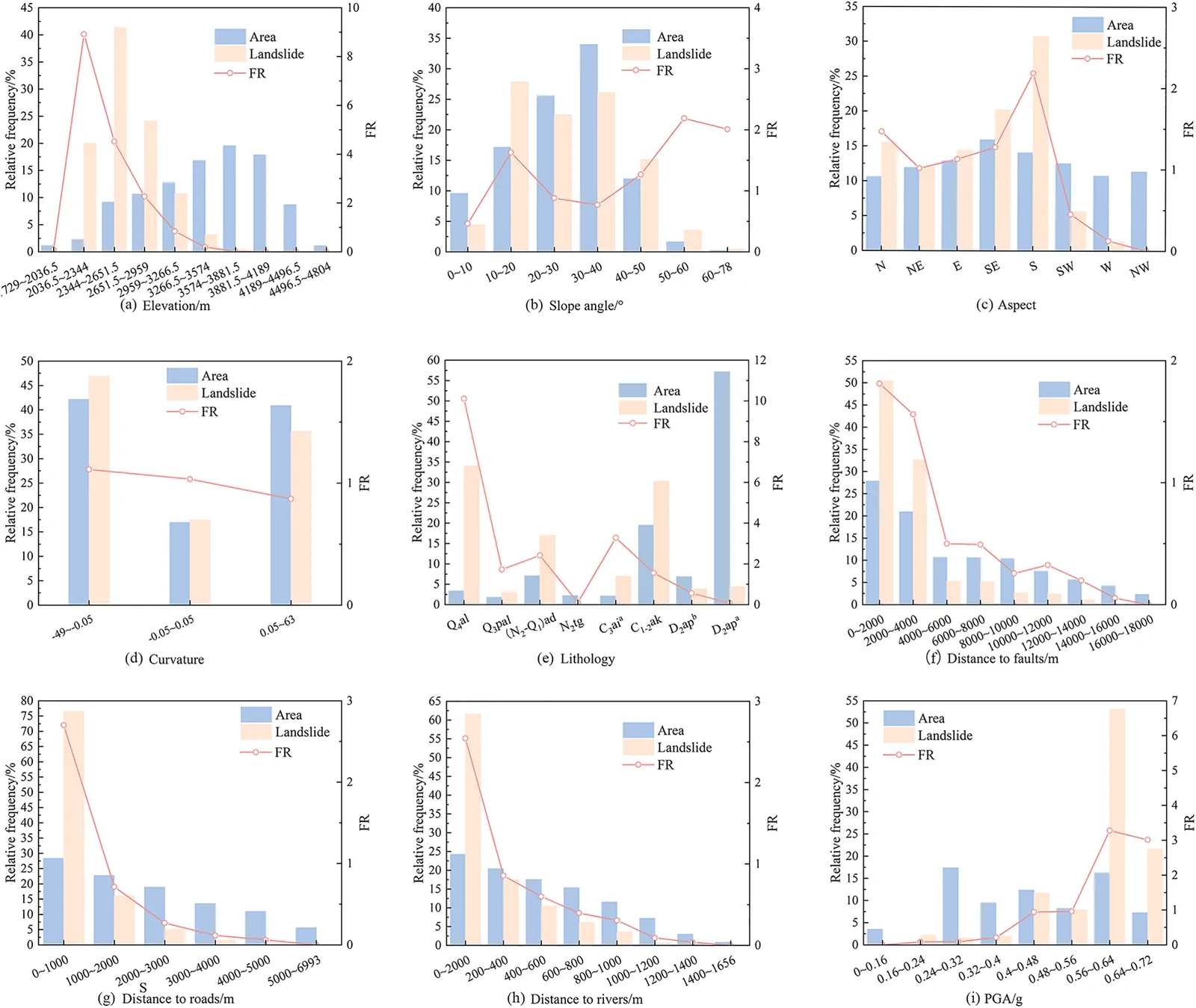
Frequency ratio values for each class of landslide conditioning factors.

Concerning slope angle, FR values exceed 1 in the classes of 10–20° (FR = 1.63), 40–50° (FR = 1.27), 50–60° (FR = 2.19), and 60–78° (FR = 2.01) (Fig 5b), indicating a high tendency for landslide occurrence. In contrast, FR values for slope classes below 10° are below 0.5, and the class of 30–40° has an FR of 0.77, indicating a lower probability of landslide occurrence.

The aspect was divided into eight directional classes (Table 1). FR values exceed 1 on north (FR = 1.46), northeast (FR = 1.01), east (FR = 1.13), and southeast (FR = 1.27)-facing slopes, indicating that slopes with these aspects are more susceptible to landsliding (Fig 5c). Of these, the FR values of northeast- (1.01) and east-facing (1.13) slopes are only marginally above 1, indicating a weak tendency rather than a strong enrichment. In addition, south-facing slopes have the highest FR value (2.18) and contain 30.8% of all landslide points, showing the most significant tendency for landslide occurrence.

For the curvature, concave slopes (FR = 1.11) have an FR value slightly greater than 1 (Fig 5d), indicating that this landform type is weakly more susceptible to landsliding. Flat slopes (FR = 1.03) also show a value marginally above 1, mainly due to small-scale landslides occurring on river terraces within the valley. These landslides often deposit material into the river channel or onto the treads of lower terraces.

With regard to lithology, landslides show high susceptibility in Q_4_al (FR = 10.11), C_3_ai^a^ (FR = 3.28), (N_2_-Q_1_)ad (FR = 2.42), Q_3_apl (FR = 1.73), and C_1-2_ak (FR = 1.56) (Fig 5e). Among them, Q_4_al has the highest FR value (10.11) and contains 34.1% of all landslide points. Loose structure and poor cementation of alluvial gravels make them prone to shallow landslides. C_3_ai^a^ (limestone and sandstone) and similar units, due to strong tectonic deformation and well-developed joints, are susceptible to rockfalls under steep slope conditions. In contrast, D_2_ap^b^ (FR = 0.57) has an FR value below 1, indicating a lower landslide probability. D_2_ap^a^ also has a low FR (0.08, n = 40). This unit covers 57.13% of the study area but contains only 4.42% of the landslide points, and its low FR may partly reflect its spatial distribution in areas of lower PGA and greater distance from faults, rather than an intrinsically higher rock-mass stability.

In terms of distance to faults, FR values decrease with increasing distance to faults (Fig 5f). FR values exceed 1 within 4,000 m of the fault, with the highest FR value (1.81) occurring in the 0–2,000 m zone, which contains 50.4% of all landslide points. Beyond 4,000 m, FR values drop below 0.5, indicating a sharp decrease in landslide probability. This distribution pattern suggests that tectonic disturbance and fractured rock masses associated with the fault zone control the spatial pattern of coseismic landslides in the study area.

For distance to rivers, FR values exceed 1 within 200 m of rivers (Fig 5g), with the highest FR value (2.54) occurring in the 0–200 m zone, which contains 61.6% of all landslide points, indicating that areas near rivers are extremely high-susceptibility zones for coseismic landslides. Beyond 200 m, FR values drop below 1 and continue to decline, falling below 0.1 beyond 1,000 m, indicating a significantly lower probability of landslide occurrence. This pattern indicates that fluvial erosion and steep valley slopes associated with river incision exert a strong control on the spatial distribution of coseismic landslides in the study area.

For distance to roads, FR values exceed 1 within 1,000 m of the Biedieli Port Highway (Fig 5h), with the highest FR value (2.70) occurring in the 0–1,000 m zone, which contains 76.5% of all landslide points, indicating that areas near the road are extremely high-susceptibility zones for coseismic landslides (Table 1). Beyond 1,000 m, FR values drop below 1 and continue to decline, falling below 0.1 beyond 3,000 m, indicating a significantly lower probability of landslide occurrence. This spatial pattern reflects the influence of road construction on slope stability, including slope cutting, excavation, and traffic vibrations, which together shape the high-risk zones distributed along the road corridor.

For PGA, FR values are below 1 for intensities up to 0.56 g, but increase sharply to 3.279 in the 0.56–0.64 g interval and 3.012 in the 0.64–0.72 g interval, respectively (Fig 5i), indicating that landslides are strongly concentrated in areas of high ground shaking. In contrast, PGA values below 0.48 g have FR values below 1, suggesting a lower probability of coseismic landsliding under weaker ground motion. This pattern confirms that seismic ground motion intensity controls the spatial distribution of coseismic landslides.

#### 4.1.2 Multicollinearity analysis.

The VIF values for the nine conditioning factors are as follows (Fig 6): PGA (7.97), elevation (6.54), distance to faults (4.61), distance to roads (2.23), distance to rivers (1.66), slope (1.46), lithology (1.39), aspect (1.03), and curvature (1.01). All VIF values are below 10, indicating that no significant multicollinearity exists among the nine conditioning factors. Therefore, all nine factors were retained for subsequent landslide susceptibility modeling.

**Fig 6:**
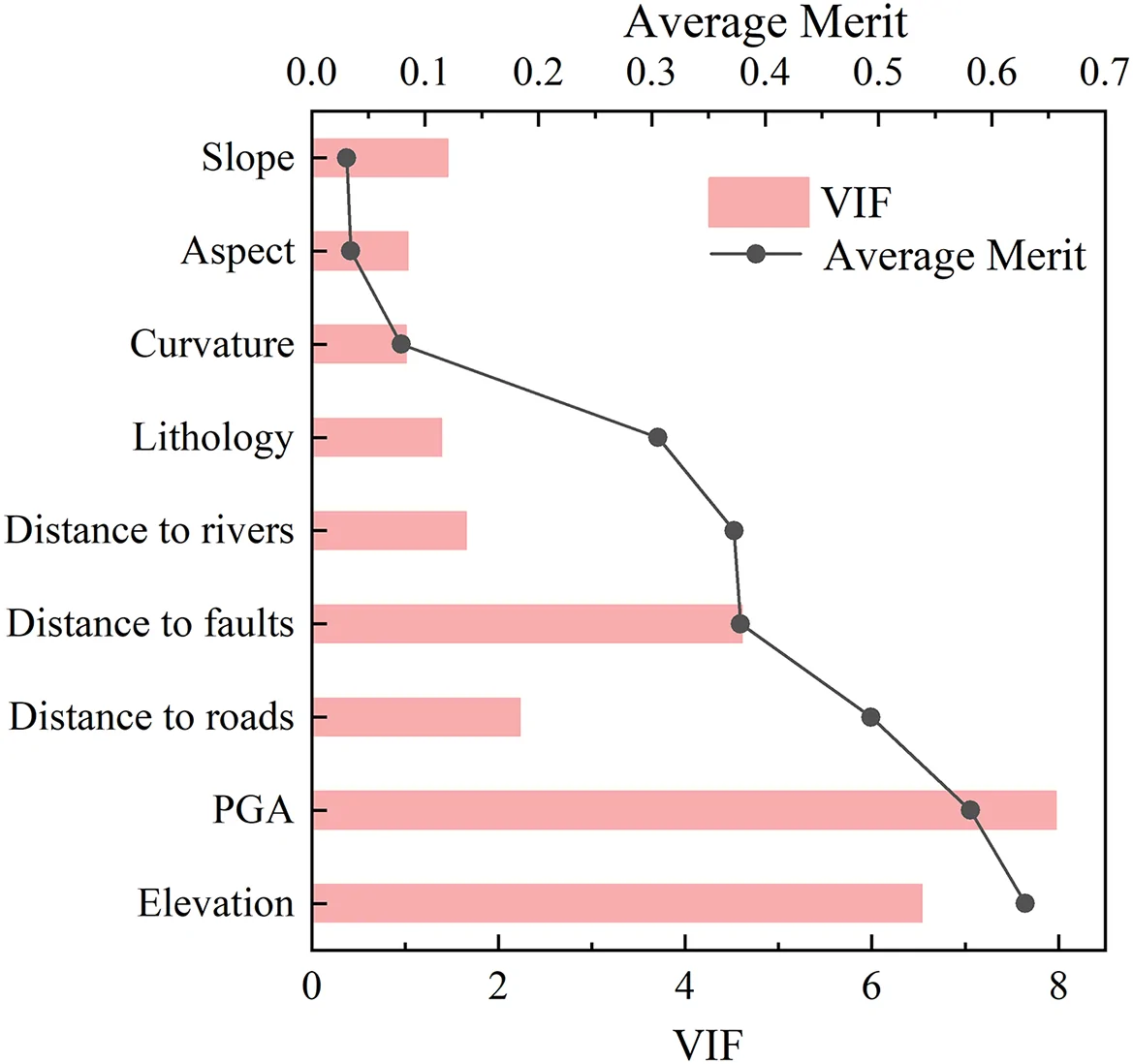
Multicollinearity analysis and importance evaluation of conditioning factors.

#### 4.1.3 Correlation attribute evaluation (CAE).

The CAE results rank the nine conditioning factors by their predictive power (Fig 6). All nine factors contribute positively to landslide susceptibility and are suitable for further analysis. Elevation had the highest average merit (AM = 0.6296), followed by PGA (AM = 0.5810), distance to roads (AM = 0.4934), distance to faults (AM = 0.3783), distance to rivers (AM = 0.3726), and lithology (AM = 0.3055). The remaining factors, curvature (AM = 0.0790), aspect (AM = 0.0345), and slope (AM = 0.0309), have relatively low AM values but still greater than zero, indicating limited but non-negligible individual predictive power.

### 4.2 Coseismic landslide susceptibility maps

The landslide susceptibility index (LSI) values calculated by the four models all ranged from 0 to 1. The equal interval method with a class width of 0.2 was used to divide the study area into five susceptibility zones: very low [0, 0.2), low [0.2, 0.4), moderate [0.4, 0.6), high [0.6, 0.8), and very high [0.8, 1.0] (Fig 7) [40]. This method is commonly used in landslide susceptibility mapping, offering the advantages of objective standards, simplicity, and transparency, which facilitate comparison across different studies. Although the susceptibility index may exhibit a skewed distribution, the equal interval method provides a stable reference framework that clearly reflects the relative level of the susceptibility index. To quantitatively analyze the coseismic landslide susceptibility map of the Biedieli River Basin, the area proportion, landslide proportion, and frequency ratio (FR) for each susceptibility zone of each model are summarized in Fig 8.

**Fig 7:**
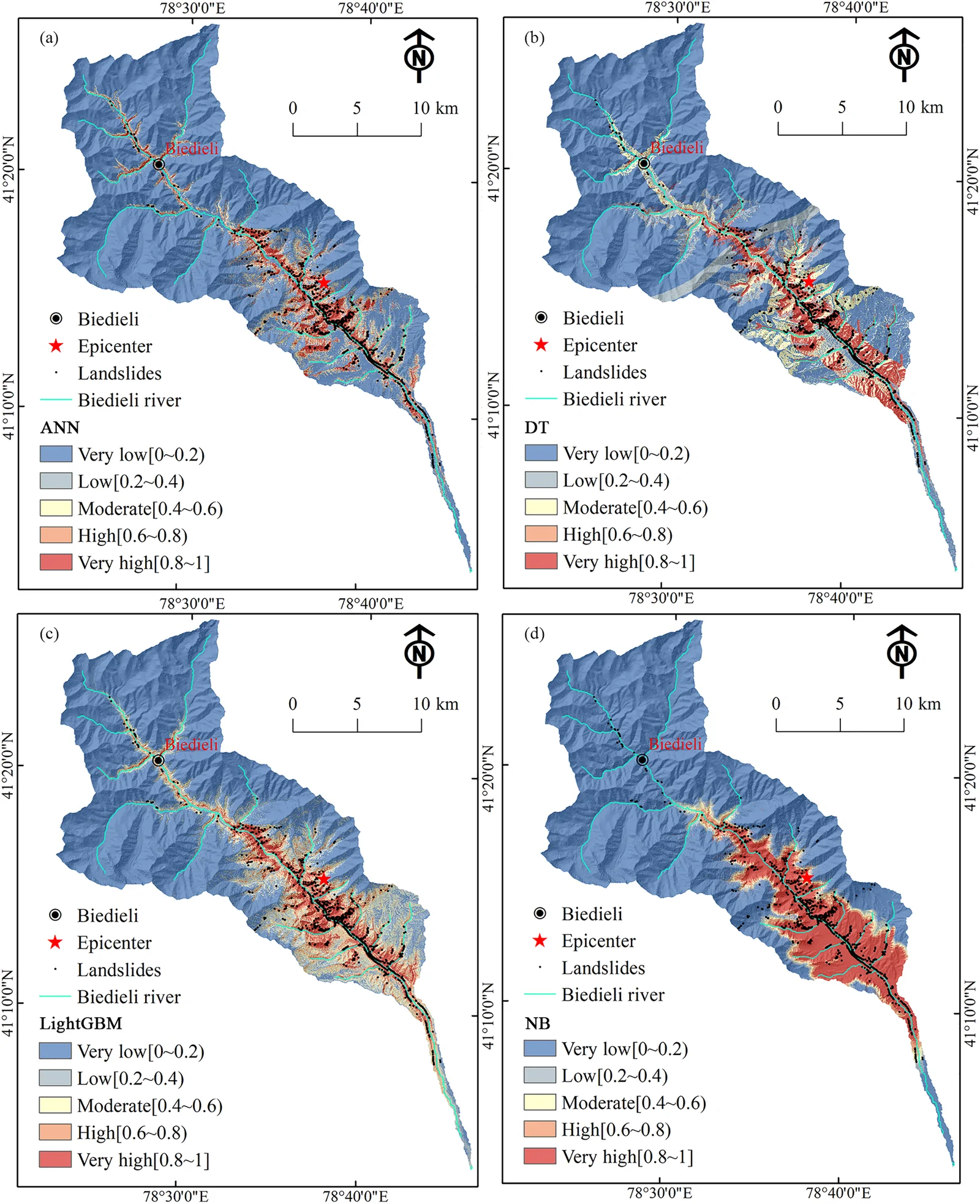
Coseismic landslide susceptibility maps based on (a) ANN, (b) DT, (c) LightGBM, and (d) NB models.

**Fig 8:**
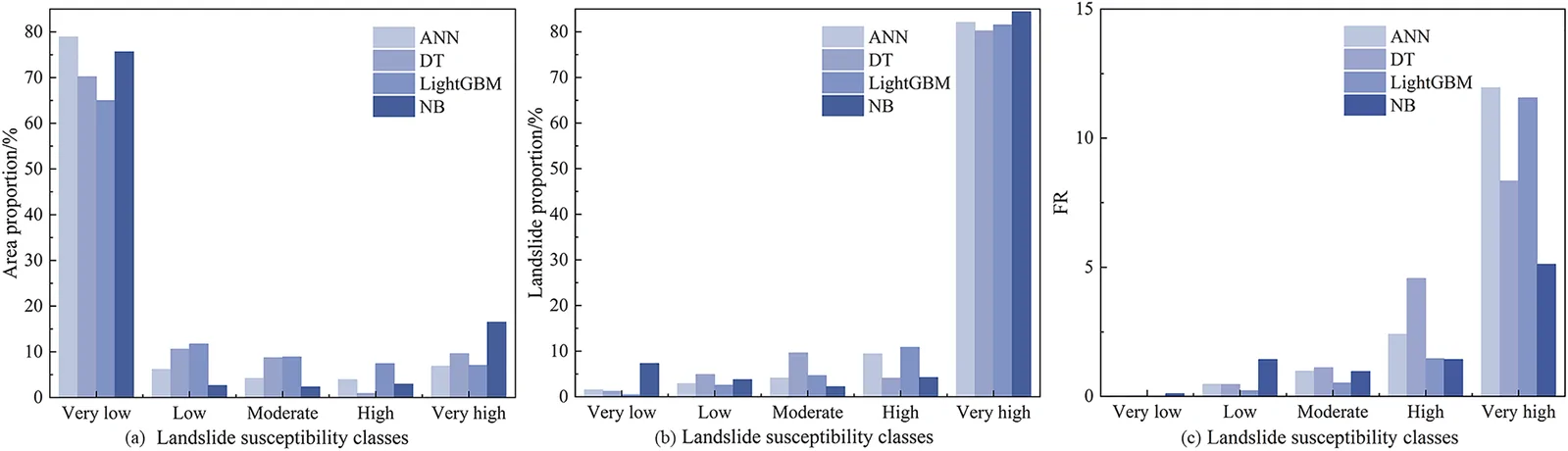
Quantitative analysis of the landslide susceptibility maps. (a) Area proportion of each susceptibility class. (b) Landslide proportion within each susceptibility class. (c) FR for each landslide susceptibility class.

In terms of area proportion, the NB model has the largest very high susceptibility zone, accounting for 16.50%, followed by DT (9.61%), LightGBM (7.05%), and ANN (6.87%). For the high susceptibility zone, LightGBM accounts for 7.41%, ANN for 3.91%, NB for 2.93%, and DT for 0.90%. The combined proportion of the very high and high susceptibility zones is smallest for DT (10.51%), followed by ANN (10.78%), LightGBM (14.46%), and largest for NB (19.43%).

For landslide proportion, the very high susceptibility zone contains the highest percentage of landslides for NB (84.40%), followed by ANN (82.08%), LightGBM (81.53%), and DT (80.20%). Combining the very high and high susceptibility zones, LightGBM captures 92.37% of all landslides, ANN captures 91.48%, NB captures 88.60%, and DT captures the lowest proportion (84.29%). In the very low and low susceptibility zones, LightGBM contains only 2.98% of landslides, ANN contains 4.43%, DT contains 6.09%, while NB contains 11.06%. These results indicate that LightGBM captures the vast majority of landslides within a small area, whereas NB retains a certain number of landslides in the low susceptibility zones, demonstrating relatively weaker discriminative ability.

With respect to frequency ratio, the FR values of ANN, DT, and LightGBM increase progressively with susceptibility level, indicating that these three models can effectively distinguish different susceptibility zones. In contrast, the FR sequence of NB is not strictly monotonic, with the FR of the low susceptibility zone (1.43) exceeding that of the moderate susceptibility zone (0.96), suggesting poorer class separation. For the very high susceptibility zone, ANN achieves the highest FR (11.95), closely followed by LightGBM (11.56), DT (8.34), and NB (5.11). The FR values of ANN and LightGBM in the very high susceptibility zone are comparable and substantially higher than those of DT and NB, demonstrating that these two models more accurately characterize the spatial clustering of landslide distribution. This finding is consistent with previous studies, which have shown that a higher FR in the very high susceptibility zone indicates a more reliable model.

In summary, the very high- and high-susceptibility zones for coseismic landslides are mainly distributed in a belt along the middle reaches of the Biedieli River valley. This spatial pattern is closely related to the Maidan Fault zone, high PGA values, and fluvial erosion. Although the four models exhibit slight differences in their zoning results, their spatial patterns are generally consistent, all identifying the middle reaches of the river valley as the very high susceptibility zone.

### 4.3 Model validation and comparison

Based on the training dataset, all four models exhibited excellent fitting capability, with AUC values exceeding 0.90 (Fig 9a). Among them, the LightGBM model performed best (AUC = 0.987), followed by ANN (AUC = 0.986), DT (AUC = 0.955), and NB (AUC = 0.907). The accuracy and F_1_-score of all models exceeded 0.83 (Fig 10a), indicating favorable agreement between model predictions and the true labels. LightGBM achieved the highest accuracy (0.942), precision (0.931), F_1_-score (0.943), and specificity (0.930), together with the lowest false positive rate (FPR = 0.071), outperforming the other three models across nearly all evaluation metrics. The ANN model attained a comparable sensitivity (0.956) and the second-highest AUC, whereas NB yielded the lowest precision (0.795) and specificity (0.770) as well as the highest FPR (0.230), reflecting a stronger tendency to misclassify non-landslide samples as landslide-prone.

**Fig 9:**
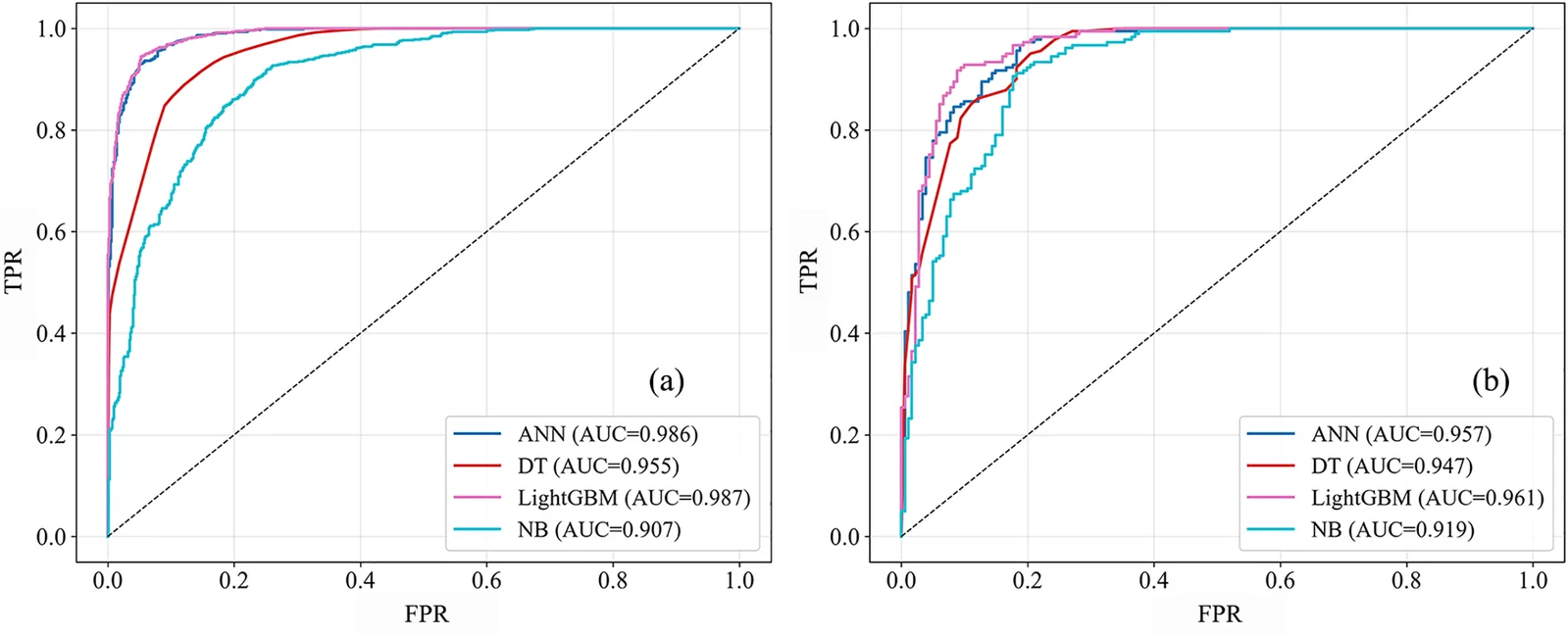
ROC curves and AUC values for the four models. (a) training dataset, (b) validation dataset.

**Fig 10:**
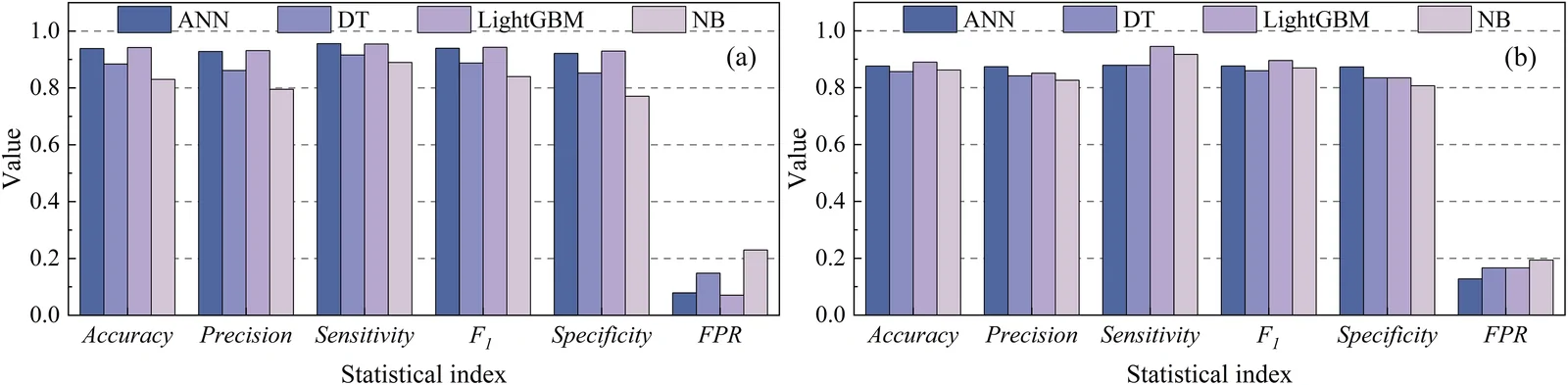
Performance comparison of the four models using statistical index. (a) training dataset, (b) validation dataset.

For the validation dataset, all four models demonstrated strong predictive capability for coseismic landslide susceptibility, with AUC values exceeding 0.91 (Fig 9b). The LightGBM model exhibited the best overall predictive performance, achieving the highest AUC (0.961), accuracy (0.890), F_1_-score (0.895), and recall (0.945), demonstrating superior discriminative accuracy and classification consistency (Fig 10b). It was followed by ANN (AUC = 0.957, accuracy = 0.876, F_1_ = 0.876), NB (AUC = 0.919, accuracy = 0.862, F_1_ = 0.869), and DT (AUC = 0.947, accuracy = 0.856, F_1_ = 0.860). Notably, ANN achieved the highest precision (0.874) and specificity (0.873) as well as the lowest FPR (0.127) on the validation dataset, indicating that its predictions produce fewer false alarms, whereas LightGBM and NB obtained higher recall at the cost of more false positives (FPR = 0.166 and 0.193, respectively).

Considering both the training and validation datasets, LightGBM consistently outperformed the other models across the majority of evaluation metrics. Its AUC decreased by only 0.026 from training to validation, and its accuracy and F_1_-score dropped by merely 0.052 and 0.047, respectively, indicating good generalization capability. ANN ranked second overall, followed by DT and NB.

## 5 Discussion

### 5.1 Dominant controlling factors and development mechanisms of coseismic landslides

In this study, nine initial conditioning factors related to topography, geology, environment, and seismicity were selected for coseismic landslide susceptibility assessment in the Biedieli River basin. Multicollinearity diagnosis was conducted using the VIF. The VIF values for all factors were below 10, indicating no significant multicollinearity issues. Thus, no factor elimination for dimension‑reduction was required. All factors yield AM values greater than 0 as calculated by CAE, which indicates that each selected factor contributes to landslide occurrence. These nine factors effectively represent the geological and environmental characteristics of the study area and reflect the multi-scale controlling mechanisms of coseismic landslide development in the Biedieli River basin [41]. No fixed threshold was adopted to remove low‑weight factors in this study. Retaining all factors avoids the loss of potentially valid information caused by over‑filtering and enables the model to comprehensively consider the synergistic effects of multiple variables.

The CAE results can be used to interpret the dominant controlling factors [30]. Elevation is the most critical factor affecting landslide occurrence (AM = 0.6296). FR analysis reveals that landslides are concentrated in the middle elevation range of 2036.5–2959 m, accounting for 85.5% of the total landslides. This elevation interval corresponds to the zone with the most intensive fluvial erosion, where river terraces are widely developed and human activities (e.g., highway construction for the Biedieli Port) are relatively concentrated. Regions with low elevation (< 2000 m) are dominated by gentle valley‑floor plains, whereas high‑elevation zones (> 3500 m) feature exposed bedrock, sparse vegetation and limited human disturbance. The middle elevation zones experience moderate seismic ground motion and the superposition of multiple hazard‑inducing factors including terraces, fluvial erosion and human activities, which makes them high‑incidence zones for coseismic landslides. This finding is consistent with previous studies [42,43], in which elevation ranked as the most important conditioning factor for coseismic landslide susceptibility. PGA is an important factor inducing landslides (AM = 0.5810). FR values remain below 1 for PGA intervals up to 0.56 g, but increase sharply to 3.279 in the 0.56–0.64 g range and 3.012 in the 0.64–0.72 g range (Fig 5i). The 0.56–0.64 g interval alone contains 53.1% of all landslide points, and the 0.64–0.72 g interval contains a further 21.7%. This distribution indicates that seismic ground motion intensity is a major controlling factor on the degree of slope instability, and that landslide density increases markedly once PGA exceeds approximately 0.56 g. This finding supports the view of Meunier et al. [44] that topographic amplification effects and local site conditions significantly influence coseismic landslide distribution. Distance to roads is the third most important factor (AM = 0.4934). FR analysis shows that 76.5% of landslide points are concentrated within 1,000 m of the Biedieli Port highway. Road construction alters the stress state of original slopes. Cutting, filling, and traffic-induced vibration all reduce slope stability. This result reflects the amplifying effect of human engineering activities on earthquake-triggered landslides. It also confirms that areas disturbed by human activities are often high-risk zones for coseismic landslides. The FR values for distance to faults are greater than 1 within 4000 m and decrease with increasing distance. Within the 0–2,000 m range, 50.4% of landslide points are concentrated. The seismogenic fault of the 2024 Wushi Ms 7.1 earthquake is the Maidan Fault, and the epicenter is located near the fault zone. Tectonic disturbance and fractured rock masses within the fault zone provide the material basis for landslide development. Meanwhile, the rupture direction of seismic motion propagates along the fault zone, resulting in a zonal distribution of landslides along the fault. The distance to rivers factor exhibits a strong control effect within the 200 m buffer zone (FR = 2.543), containing 61.6% of landslide points. Lateral fluvial erosion undermines toe support and creates free faces favourable for landsliding, whilst river incision increases relative relief and gravitational potential energy of slopes. Landslides are predominantly distributed in belts on both sides of the middle‑reach valley of the Biedieli River, and this spatial pattern is closely associated with fluvial erosion. In terms of lithology (AM = 0.3055), the FR value of Q_4_al (Quaternary alluvial gravel) reaches 10.11, containing 34.1% of landslide points. Such loose deposits possess porous texture and poor cementation. They constitute the material of river terraces and are highly susceptible to instability under seismic shaking. The FR values of C_3_ai^a^ (limestone and sandstone) and C_1-2_ak (sandstone, siltstone, limestone, and gypsum) are also greater than 1, indicating that sedimentary rock areas are similarly prone to landslides. In contrast, metamorphic rocks such as D_2_ap^a^ (phyllite, siltstone, and limestone) show a low FR value of only 0.08 and exhibit relatively high stability.

From a disaster prevention and mitigation perspective, the CAE-based ranking provides a practical basis for prioritizing monitoring and engineering interventions. Elevation and PGA jointly define the priority zones for post-earthquake emergency response, suggesting that rapid ShakeMap-based PGA estimates could be used to quickly delineate the most hazardous belt and guide the deployment of rescue and inspection teams. The road corridor requires prioritized slope investigation, drainage maintenance, and engineering stabilization, given that road construction and fluvial erosion jointly disturb the valley-floor slopes. Fault-adjacent slopes should be included in routine monitoring programs, and infrastructure siting within the fault zone should be accompanied by site-specific seismic hazard assessment. Riverbank slopes and terrace scarps along the middle reaches of the Biedieli River should be treated as key monitoring segments, because they are susceptible to both coseismic and post-seismic instability. Finally, construction activities on Q_4_al terrace deposits should be accompanied by drainage control and slope protection measures. Taken together, these findings translate the statistical ranking of controlling factors into spatially explicit and factor-specific mitigation strategies for the Biedieli River basin.

In summary, PGA and elevation control the dynamic conditions and spatial distribution of coseismic landslides. Distance to roads and distance to faults reflect the influences of human engineering activities and tectonic disturbance on slope stability, respectively. Lithology provides the material basis for landslide initiation, whereas distance to rivers represents the modification of slope‑toe stress state induced by lateral fluvial erosion. These factors jointly control the development pattern of coseismic landslides in the Biedieli River basin and provide the basis for the factor-specific mitigation strategies outlined above.

### 5.2 Model reliability

The ROC curve and statistical index analyses indicate that all four machine learning models exhibit good performance in coseismic landslide susceptibility assessment, with the predictive capability ranked as LightGBM > ANN > DT > NB. Certain discrepancies exist in prediction accuracy, robustness and generalization ability among the four models, which are related to their respective algorithm mechanisms and geo‑environmental characteristics of the study area. It is worth noting that the dataset comprises 1,808 samples (904 landslide and 904 non-landslide points) against only nine conditioning factors, yielding a high sample-to-feature ratio that supports stable model training. Moreover, the performance decay from the training to the validation dataset is minor for all four models (AUC degradation ≤ 2.9%), indicating that the sample size is sufficient for both the single models and the ensemble model, without signs of underfitting caused by data scarcity.

The LightGBM model exhibits the optimal overall performance, yielding the highest AUC, accuracy (0.942, 0.890) and F_1_‑score (0.943, 0.895) on both the training dataset (AUC = 0.987) and validation dataset (AUC = 0.961). This is mainly attributed to its ensemble‑learning mechanism. A sequence of decision trees is constructed iteratively under the gradient‑boosting framework, where each new tree focuses on correcting the residuals of the previous tree. Accordingly, the model can continuously target hard‑to‑classify samples and substantially improve its capacity to capture complex nonlinear relationships. Meanwhile, LightGBM adopts a leaf-wise growth strategy and GOSS sampling technique, which enables efficient processing of high‑dimensional features and effective overfitting mitigation [45]. Most importantly, this model presents minor performance degradation on the validation dataset (AUC decreases from 0.987 to 0.961, with a degradation of only 2.6%). The achieved performance is comparable to that reported in similar studies. For the 2017 Jiuzhaigou earthquake-affected area, the best-performing model (XGBoost) obtained a validation AUC of 0.910, and LightGBM yielded 0.897 [25]; for the 2015 Mw 7.8 Gorkha earthquake, LightGBM achieved a testing AUC of 0.972 [46]. The validation AUC of LightGBM in this study (0.961) falls within this range. Moreover, the FR value for the extremely high‑susceptibility zone reaches 11.56, second only to that of ANN (11.95) and markedly higher than those of DT (8.34) and NB (5.11). In addition, the very high and high susceptibility zones of LightGBM capture 92.37% of all landslides, the highest among the four models, while only 2.98% of landslides fall within its very low and low susceptibility zones. These features highlight its low overfitting risk and outstanding generalization capacity, making it highly suitable for study areas with complex geo‑environments and highly concentrated landslide distribution such as the Biedieli River basin. This result confirms the expectation that LightGBM would balance accuracy and overfitting control under a moderately sized, highly clustered dataset, although its internal decision logic remains difficult to visualise at the local scale, consistent with the anticipated limitation in interpretability.

The ANN has strong nonlinear fitting capability and can capture the complex nonlinear relationships between landslide occurrence and various influencing factors [47]. However, the ANN model in this study adopts a simple structure with a single hidden layer of 64 neurons. In terms of performance decay from the training dataset to the validation dataset, ANN shows a small decay (AUC from 0.986 to 0.957, a decay of 2.9%), indicating good generalization ability. Nevertheless, its absolute performance remains lower than that of LightGBM. This pattern is closely related to the model architecture: a single hidden layer with 64 neurons has limited capacity to represent the high-order nonlinear interactions among factors in the study area. For example, the interaction between PGA and elevation, or between distance to rivers and lithology. Such limited capacity explains why ANN generalises well but cannot reach the absolute accuracy of LightGBM. Notably, ANN yields the highest FR (11.95) in the very high susceptibility zone. However, this value is closely tied to its small area proportion of this zone (6.87%, the smallest among the four models), because a smaller denominator mechanically inflates the FR. This therefore reflects a more compact zoning pattern rather than a decisive advantage over LightGBM. These results partially confirm the expectation for ANN. The small decay suggests that a simple network can generalise reasonably well under limited samples, whereas the lower absolute accuracy points to the capacity limit of the single-hidden-layer architecture. Whether a deeper network would improve performance without overfitting remains to be tested.

The DT model has the advantages of simple structure, high transparency, and easy interpretability. Notably, DT exhibits the smallest performance decay from the training dataset to the validation dataset (AUC from 0.955 to 0.947, a decay of only 0.8%), reflecting the stability of its simple tree structure. However, its absolute performance is the second lowest among the four models (validation accuracy = 0.856, F_1_-score = 0.860). Its “piecewise constant” prediction approach makes it difficult to smoothly fit continuous landslide susceptibility surfaces. This limitation is amplified in the Biedieli River basin because the landslide susceptibility index varies continuously with elevation and PGA, and landslides are highly clustered within a narrow middle-elevation belt. A piecewise-constant model must approximate this continuous variation with a limited number of rectangular partitions, which explains why DT produced a much larger very high susceptibility zone (9.61% versus 7.05% for LightGBM) with a lower FR (8.34), indicating over-partitioning rather than genuine enrichment of landslides. It is also sensitive to data noise, resulting in relatively limited generalization ability. These results confirm the expectation that DT would provide a transparent baseline but suffer from over-partitioning and poor landslide enrichment.

As a probabilistic classifier based on Bayesian theorem, NB theoretically offers advantages such as high computational efficiency and good adaptability to small sample data [48]. However, its performance in this study is relatively poor. The reasons are tied to the data characteristics of the study area. First, the conditional-independence assumption is violated: elevation, PGA, distance to faults, and distance to rivers are strongly collinear (e.g., VIF = 7.97 for PGA, 6.54 for elevation). NB therefore overestimates landslide probability where only one correlated factor is unfavorable, producing a diffuse susceptibility pattern. Second, the skewed distributions of PGA and elevation are over-smoothed by kernel density estimation, blurring the boundary between high and low susceptibility zones. These effects explain why NB yielded the lowest FR in the very high susceptibility zone (5.11), despite being the most computationally efficient. These results confirm the expectation that the conditional-independence assumption would not hold for the correlated conditioning factors in the study area. The low FR value in the very high susceptibility zone (5.11, compared with 11.56 for LightGBM) indicates that NB failed to concentrate landslides into a small high-risk area, limiting its practical value for susceptibility mapping despite its theoretical efficiency.

In summary, LightGBM, as an ensemble learning model, combines multiple weak decision trees through the Boosting framework and effectively improves generalization ability, thus performing the best. ANN and DT, as single models, provide reliable performance benchmarks. NB fails to fully leverage its theoretical advantages in this study because it cannot capture interactions among factors and its probability estimation for continuous factors may be biased. This comparison provides a reference for model selection in coseismic landslide susceptibility modeling under similar geological environments.

### 5.3 Coseismic landslide susceptibility zoning analysis

Comparison of the landslide susceptibility maps generated by the four models reveals that the zoning results of LightGBM and ANN are generally consistent in spatial distribution. However, the distribution of high and very high susceptibility zones in DT and NB differs from that of the former two models (Fig 7). Overall, the zoning results of the ensemble learning model (LightGBM) are more consistent with the spatial distribution of regional geological and environmental factors. The area proportions of high and very high susceptibility zones calculated by the four models are as follows: NB (19.43%), LightGBM (14.46%), ANN (10.78%), and DT (10.51%). These zones are mainly concentrated in the middle-elevation valley areas (2036.5–2959 m), steep slopes with gradients greater than 40°, areas with PGA exceeding 0.48 g, and the vicinity of the Maidan fault zone. In particular, within 4000 m of the seismogenic fault and along both sides of the main channel of the Biedieli River and its tributaries, the rock mass is fractured and slopes are steep, resulting in a high concentration of coseismic landslides. As a distinctive geomorphic unit in the study area, terraces form stepped topography composed of flat terrace surfaces and steep terrace risers. This morphology is prone to stress concentration under seismic ground motion. In addition, the terrace deposits (Q_4_al) have loose structures and poor cementation, which further exacerbate landslide occurrence. The area proportions of very low susceptibility zones calculated by the four models are as follows: ANN (78.88%), NB (75.63%), DT (70.20%), and LightGBM (64.97%), which are mainly distributed in the middle and upper reaches of the high mountain areas. These regions are characterized by high elevation, intact bedrock, limited human activity, and distance from the epicenter, where landslides are essentially absent.

In summary, LightGBM produces the most accurate zoning results, which can capture the largest proportion of landslides (92.37%) within a limited high and very high susceptibility area (14.46%), while only 2.98% of landslides fall within its very low and low susceptibility zones, the lowest among the four models, followed by ANN (91.48% of landslides within 10.78% of the area). DT and NB exhibit relatively low zoning accuracy: DT captures the smallest proportion of landslides (84.29%) in the high and very high susceptibility zones, whereas NB, despite placing 84.40% of landslides in the very high susceptibility zone, delineates an excessively large very high susceptibility area (16.50%) and retains 11.06% of landslides in the very low and low susceptibility zones, indicating a diffuse zoning pattern. It is recommended that LightGBM should be preferentially adopted for landslide susceptibility assessment in the Biedieli River basin and regions with analogous geo‑environmental settings.

In practice, the resulting susceptibility map can directly support disaster mitigation in the study area. The high and very high susceptibility zones, which are concentrated near the seismogenic fault and along the Biedieli River, should be prioritized for post-earthquake emergency investigation, landslide monitoring, and early-warning deployment, while engineering facilities and reconstruction projects located within these zones should adopt slope protection and drainage measures.

### 5.4 Limitations and perspectives

Although this study advances the application of machine learning and ensemble learning methods in coseismic landslide susceptibility mapping, several limitations remain. First, the completeness of the landslide inventory is limited. A total of 904 coseismic landslides were identified based on high-resolution remote sensing images (Jilin-1, Gaofen-7, and Gaofen-2). However, due to image resolution constraints and shadow occlusion, some small-scale rockfalls and shallow debris landslides may have been omitted. In addition, field validation did not cover all landslide points because of adverse weather conditions (snowfall) and aftershocks following the earthquake. Future studies should integrate higher-resolution remote sensing images (e.g., 0.5 m resolution) and unmanned aerial vehicle (UAV) aerial data to further supplement omitted landslides and improve inventory completeness.

Second, the strategy used to generate negative samples may introduce bias. In this study, non-landslide samples were randomly generated in areas outside the landslide polygons, without applying a fixed buffer distance from the landslide boundaries, without matching the terrain conditions of the landslide samples, and without controlling for spatial autocorrelation. This approach carries the risk of mislabelling potentially unstable but unidentified or obscured areas as negative samples, which may cause the model to underpredict susceptibility in some high-risk zones. In addition, because the training and validation samples were randomly partitioned, nearby samples may share similar terrain conditions, and the validation performance may therefore be somewhat optimistic. This limitation stems from the inherent difficulty of identifying hidden or partially obscured slope instabilities from remote sensing imagery alone. Although the randomly generated non-landslide points were visually checked and those located in obviously unstable or suspicious positions were excluded, a systematic spatial constraint was not implemented. Future studies could define non-landslide areas by applying a spatial buffer around landslide polygons, adopt terrain-matched sampling and spatially blocked validation, or incorporate interferometric synthetic aperture radar (InSAR)-derived deformation information to refine negative-sample selection, thereby improving the model’s discriminative capability.

Third, this study selected nine factors related to topography, geology, environment, and seismicity for coseismic landslide susceptibility assessment, but did not individually consider dynamic environmental factors such as freeze-thaw cycles, rainfall, and groundwater that can influence long-term slope stability. The Biedieli River basin is located in a mid‑temperate plateau arid climate zone with large diurnal temperature variations. Freeze‑thaw processes exert a significant pre‑weakening effect on rock weathering and fracturing, further reducing the critical instability threshold of slopes under seismic loading. Future studies could further incorporate such dynamic environmental factors and construct time‑variant landslide susceptibility models to more comprehensively reveal the development mechanisms of coseismic landslides under the coupling of environmental pre‑weakening and seismic triggering.

Fourth, the interpretability of machine learning models remains limited. Although LightGBM achieves the best predictive performance, its internal mechanism is like a “black box”. It is difficult to explicitly quantify the contribution of each conditioning factor to landslide susceptibility at the local scale. In this study, the CAE-based importance ranking and the FR analysis partially offset this limitation, as they quantify the overall contribution of each factor and identify its susceptible ranges. However, both methods provide dataset-level statistics: they describe the average effect of each factor across all samples, but cannot quantify how much each factor contributes to a specific prediction, nor reveal interactions among factors within individual predictions. This limitation was not addressed in the present study, primarily because the study is designed as a comparative assessment of four models rather than an in-depth interpretability study of a single model. Future studies could employ explainable artificial intelligence methods, such as SHAP (Shapley Additive exPlanations) and partial dependence plots (PDP), to interpret model outputs and quantify factor contributions at the individual prediction level, to examine the nonlinear thresholds suggested by the FR analysis, and to translate the factor ranking into marginal-effect curves that can directly inform factor-specific mitigation strategies, thereby enhancing model transparency and credibility.

Fifth, the model comparison was based on a single random partition, without repeated runs or statistical significance testing. The validation AUC difference between LightGBM and ANN is marginal (0.004), and their ranking may vary under different partitions. The preference for LightGBM therefore rests on its higher accuracy, F_1_-score, and landslide capture rate rather than only on the AUC difference; repeated experiments with statistical testing are left for future work.

Despite the above limitations, this study still demonstrates the effectiveness of ensemble learning in coseismic landslide susceptibility mapping in the Biedieli River basin. Future research could also consider integrating multi-source data, such as InSAR and unmanned aerial vehicle (UAV), as well as physics-informed machine learning methods, to further improve prediction accuracy and model interpretability under different scenarios.

## 6 Conclusions

This study takes the coseismic landslides in the Biedieli River basin triggered by the 2024 Wushi Ms 7.1 earthquake as the research object and compares the susceptibility prediction performance of three single machine learning models (ANN, DT, and NB) and one ensemble learning model (LightGBM). The main conclusions are as follows:

(1) A total of 904 coseismic landslides were identified in the Biedieli River basin, with a total area of 2.65 km^2^. The area of individual landslides ranges from 54.32 m^2^ to 74,360.25 m^2^, with an average area of 2,928.84 m^2^. Landslides are mainly distributed in belts along both sides of the middle‑reach valley of the Biedieli River and concentrated near the Maidan Fault zone, with the epicentral area falling within this high‑density distribution zone. The landslides are dominated by shallow debris landslides, rockslides and rock falls.
(2) Elevation (AM = 0.6296), PGA (AM = 0.5810), and distance to roads (AM = 0.4934) are the most important factors affecting landslide occurrence. FR analysis further reveals the optimal ranges for each factor: elevation within 2,036.5–2,344 m (FR = 8.91) and PGA within 0.48–0.56 g (FR = 6.50) yield the highest FR values. For anthropogenic and tectonic factors, distance to roads within 0–1,000 m (FR = 2.70) and distance to faults within 0–2,000 m (FR = 1.81) show relatively high landslide frequency. In terms of lithology, Q_4_al (Quaternary alluvial gravel) exhibits the highest FR value (10.11). VIF analysis indicates that all factors have no significant multicollinearity.
(3) Among the four models, LightGBM achieves the best predictive performance (validation dataset AUC = 0.961, accuracy = 0.890%), followed by ANN (AUC = 0.957), DT (AUC = 0.947), and NB (AUC = 0.919). As an ensemble learning model, LightGBM effectively improves generalization ability through the Boosting framework, with a performance decay of only 2.6%. It captures 81.53% of the total landslides within its very high susceptibility zone, which occupies only 7.05% of the total area, and 92.37% of all landslides within the combined high and very high susceptibility zones, demonstrating low overfitting and excellent generalization capability. Accordingly, it is suggested that LightGBM be preferentially adopted for coseismic landslide susceptibility assessment in the Biedieli River Basin and regions with analogous geo‑environmental conditions.
(4) The high‑ and very high‑susceptibility zones are predominantly distributed in middle‑elevation river valleys (2036.5–2959 m), steep slopes with gradient > 40°, areas with PGA > 0.48 g and the vicinity of the Maidan fault zone, showing high spatial consistency with terrace distribution. Terraces form stepped landforms consisting of flat terrace surfaces and steep terrace risers, where stress concentration readily occurs under seismic loading. Combined with the loose texture and weak cementation of terrace deposits (Q_4_al), slope failure is further promoted.

## Supporting information

S1 File Training dataset. (CSV)
